# Supplementary material for: Maturation of striatal dopamine supports the development of habitual behavior through adolescence
Source: bioRxiv. 2025 Jan 6:2025.01.06.631527. Preprint. [Version 1] doi: 10.1101/2025.01.06.631527 (PMC11741407; doi:10.1101/2025.01.06.631527)
Supplement: 1 [file NIHPP2025.01.06.631527V1-supplement-1.pdf]

bioRxiv preprint doi: https://doi.org/10.1101/2025.01.06.631527; this version posted January 6, 2025. The copyright holder for this preprint (which was not certified by peer review) is the author/funder, who has granted bioRxiv a license to display the preprint in perpetuity. It is made available under aCC-BY 4.0 International license.

Supplementary Materials

S.1. Supplementary results from section 3.2

Detailed summaries from GAMMs assessing developmental trajectories of tissue iron in the basal ganglia are presented in Supplementary Tables S1. All age smooths are significantly different than 0 at Bonferroni-adjusted α of 0.0125.

Table S1. GAMM results characterizing developmental trajectories of nT2*w.

ROI					
**Pallidum**	**Parametric coefficients**				
		Estimate	S.E.	*t*	*p*
	Intercept	0.58	0.01	89.48	< 0.001*
	Sex	-0.004	0.01	-0.64	0.52
	Visit	-0.004	0.003	-1.09	0.28
	**Approx. significance of smooth terms**				
		edf	Ref. edf	*F*	*p*
	Age	1.21	1.29	43.68	< 0.001*
	**Random effects**				
			sd		95% CI
	Intercept		0.04		[0.03, 0.05]
	Residuals		0.03		[0.03, 0.04]
**Caudate**	**Parametric coefficients**				
		Estimate	S.E.	*t*	*p*
	Intercept	0.87	0.01	94.91	< 0.001*
	Sex	0.002	0.01	0.18	0.86
	Visit	0.03	0.005	5.62	< 0.001*
	**Approx. significance of smooth terms**				
		edf	Ref. edf	*F*	*p*
	Age	1.92	1.96	5.94	0.003*
	**Random effects**				
			sd		95% CI
	Intercept		0.06		[0.05, 0.07]
	Residuals		0.05		[0.04, 0.06]

ROI					
**NAcc**	**Parametric coefficients**				
		Estimate	S.E.	*t*	*p*
	Intercept	0.88	0.02	48.14	< 0.001*
	Sex	-0.04	0.02	-2.15	0.03
	Visit	0.03	0.01	2.69	0.007*
	**Approx. significance of smooth terms**				
	edf	Ref. edf	*F*	*p*	
	Age	1	1	18.69	< 0.001*
	**Random effects**				
			sd		95% CI
	Intercept		0.12		[0.10, 0.14]
	Residuals		0.09		[0.08, 0.11]
**Putamen**	**Parametric coefficients**				
		Estimate	S.E.	*t*	*p*
	Intercept	0.88	0.01	117.81	< 0.001*
	Sex	-0.03	0.01	-3.18	0.002*
	Visit	0.02	0.004	4.16	< 0.001*
	**Approx. significance of smooth terms**				
		edf	Ref. edf	*F*	*p*
	Age	1	1	87.16	< 0.001*
	**Random effects**				
			sd		95% CI
	Intercept		0.05		[0.04, 0.06]
	Residuals		0.038		[0.03, 0.04]

Note. Edf = effective degrees of freedom. * *p* is significant at Bonferroni-adjusted α of 0.0125

bioRxiv preprint doi: https://doi.org/10.1101/2025.01.06.631527; this version posted January 6, 2025. The copyright holder for this preprint (which was not certified by peer review) is the author/funder, who has granted bioRxiv a license to display the preprint in perpetuity. It is made available under aCC-BY 4.0 International license.

S.2. Supplementary results from section 3.3.

Detailed summary tables from GAMMs assessing developmental trajectories of behavioral measures during the two-stage sequential decision-making task are presented in Supplementary Table S2. All age smooths are significantly different than 0.

Table S2. GAMM results characterizing developmental trajectories of behavioral measures.

Behavioral measure	Parametric coefficients				
		Estimate	S.E.	*t*	*p*
	Intercept	1.12	0.11	10.21	< 0.001*
	Sex	0.37	0.12	3.11	0.002*
	Visit	0.10	0.06	1.68	0.09
	**Approx. significance of smooth terms**				
**First-stage stay**		*edf*	Ref. edf	*F*	*p*
	Age	1.71	1.81	40.13	< 0.001*
	**Random effects**				
		sd		95% CI	
	Intercept	0.70		[0.60, 0.81]	
	Residuals	0.57		[0.50, 0.65]	
	**Parametric coefficients**				
		Estimate	S.E.	*t*	*p*
	Intercept	0.24	0.05	5.19	< 0.001*
	Sex	0.12	0.05	2.60	0.01*
	Visit	0.07	0.03	2.66	0.01*
**Model-based**	**Approx. significance of smooth terms**				
		edf	Ref. edf	*F*	*p*
	Age	1.49	1.63	15.29	< 0.001*
	**Random effects**				
		sd		95% CI	
	Intercept	0.24		[0.20, 0.30]	
	Residuals	0.27		[0.23, 0.30]	
	**Parametric coefficients**				
		Estimate	S.E.	*t*	*p*
	Intercept	0.30	0.03	10.00	< 0.001*
	Sex	0.03	0.03	1.20	0.23
	Visit	0.03	0.02	1.69	0.09
**Model-free**	**Approx. significance of smooth terms**				
		edf	Ref. edf	*F*	*p*
	Age	1.13	1.20	52.45	< 0.001*
	**Random effects**				
		sd		95% CI	
	Intercept	0.13		[0.097, 0.17]	
	Residuals	0.19		[0.17, 0.21]	

Note. Edf = effective degrees of freedom. * *p* is significant at Bonferroni-adjusted α of 0.017

bioRxiv preprint doi: https://doi.org/10.1101/2025.01.06.631527; this version posted January 6, 2025. The copyright holder for this preprint (which was not certified by peer review) is the author/funder, who has granted bioRxiv a license to display the preprint in perpetuity. It is made available under aCC-BY 4.0 International license.

S.3. Supplementary results from section 3.4.

We compared a set of four models for each ROI using an analysis of deviance test and AIC comparisons (Table S3). For the caudate, NAcc, and globus pallidus, the analysis of deviance tests favored the simpler model with just a smooth term for age. For caudate and NAcc, the lowest AIC index was for model 1. For globus pallidus, the lowest AIC was for model 2. Thus, we did not retain any of these candidate models for further comparisons.

Table S3. AIC and analysis of deviance test for caudate, NAcc, and globus pallidus

ROI	Model	AIC	Resid. DF	Resid. Dev	DF	Deviance	*F*	*p*
	**Model 1**	**-898.22**	128.19	0.41				
**Caudate**	Model 2	-895.60	126.64	0.41	1.56	-0.004		
	Model 3	-882.98	126.44	0.42	0.20	-0.003		
	Model 4	-892.51	125.53	0.41	0.91	0.002	0.89	0.34
	**Model 1**	**-495.71**	122.55	1.44				
**NAcc**	Model 2	-495.37	120.57	1.43	1.98	0.006	0.33	0.72
	Model 3	-493.82	119.97	1.44	0.60	-0.001		
	Model 4	-492.15	119.40	1.44	0.59	-0.003		
	Model 1	-1126.39	122.23	0.19				
**Globus**	**Model 2**	**-1129.54**	121.01	0.18	1.22	0.004	3.02	0.08
**Pallidus**	Model 3	-1128.94	120.19	0.18	0.82	0.0007	0.73	0.37
	Model 4	-1128.41	117.24	0.18	2.95	0.0008	0.26	0.85
	Model 1	-1046.37	123.68	0.24				
**Putamen**	Model 2	-1048.81	122.88	0.24	0.80	0.003	3.16	0.09
	**Model 3**	**-1060.50**	120.23	0.22	2.65	0.01	3.94	0.01*
	Model 4	-1058.09	119.85	0.23	0.38	-0.001		

Note. The lowest AIC fit index are bolded. AIC = Akaike information criterion. DF = Degrees of freedom for *F*-test, which reflect differences between degrees of freedom between models being compared. * = $p < 0.05$. For globus pallidus, the deviance test between model 1 and model 2 was not significant, indicating the model 1 was the better model.

Detailed summary tables from GAMM fits during model comparisons are presented in Supplementary Tables S4 – S7. For caudate (Table S5), NAcc (Table S6), and globus pallidus (Table S7), all age smooths were significant. However, no smooths from any of the behavioral measures were significant in the more complex models. For the putamen (Table S4), models 3 and 4 had significant smooths for age, first-stage stay, and model-based behavior.

bioRxiv preprint doi: https://doi.org/10.1101/2025.01.06.631527; this version posted January 6, 2025. The copyright holder for this preprint (which was not certified by peer review) is the author/funder, who has granted bioRxiv a license to display the preprint in perpetuity. It is made available under aCC-BY 4.0 International license.

Table S4. Full GAMM results of model comparisons for putamen.

Putamen										
**Model 1**	**Parametric coefficients**					**Model 2**	**Parametric coefficients**			
		Estimate	S.E.	*t*	*p*		Estimate	S.E.	*t*	*p*
	Intercept	0.88	0.01	117.91	< 0.001***	Intercept	0.87	0.01	116.36	< 0.001***
	Sex	-0.03	0.01	-3.19	0.002**	Sex	-0.02	0.01	-2.89	0.004**
	Visit	0.02	0.004	4.15	< 0.001***	Visit	0.02	0.004	4.31	< 0.001***
	**Approx. significance of smooth terms**					**Approx. significance of smooth terms**				
		edf	Ref. edf	*F*	*p*		edf	Ref. edf	*F*	*p*
	Age	1	1	87.68	< 0.001***	Age	1	1	87.73	< 0.001***
	FSS	-	-	-	-	FSS	1	1	2.40	0.12
	MB	-	-	-	-	MB	-	-	-	-
	MF	-	-	-	-	MF	-	-	-	-
	**Random effects**					**Random effects**				
		sd		95% CI			*sd*		95% CI	
	Intercept	0.046		[0.039, 0.054]		Intercept	0.046		[0.039, 0.054]	
	Residuals	0.038		[0.033, 0.043]		Residuals	0.037		[0.032, 0.043]	
**Model 3**	**Parametric coefficients**					**Model 4**	**Parametric coefficients**			
		Estimate	S.E.	*t*	*p*		Estimate	S.E.	*t*	*p*
	Intercept	0.87	0.01	116.72	< 0.001***	Intercept	0.87	0.01	116.29	< 0.001***
	Sex	-0.02	0.01	-2.95	0.004**	Sex	-0.02	0.01	-2.91	0.004**
	Visit	0.02	0.004	4.16	< 0.001***	Visit	0.02	0.004	4.15	< 0.001***
	**Approx. significance of smooth terms**					**Approx. significance of smooth terms**				
		edf	Ref. edf	*F*	*P*		*edf*	Ref. edf	*F*	*p*
	Age	1	1	86.72	< 0.001***	Age	1	1	86.73	< 0.001***
	FSS	1	1	6.21	0.01*	FSS	1	1	5.81	0.02*
	MB	1	1	4.14	0.04*	MB	1	1	3.67	0.06
	MF	-	-	-	-	MF	1	1	0.19	0.66
	**Random effects**					**Random effects**				
		sd		95% CI			*sd*		95% CI	
	Intercept	0.046		[0.039, 0.054]		Intercept	0.046		[0.039, 0.054]	
	Residuals	0.036		[0.032, 0.042]		Residuals	0.036		[0.032, 0.042]	

Note. Edf = effective degrees of freedom. FSS = first-stage stay. MB = model-based. MF = model-free. * *p* is significant at Bonferroni-adjusted α of 0.0125.

bioRxiv preprint doi: https://doi.org/10.1101/2025.01.06.631527; this version posted January 6, 2025. The copyright holder for this preprint (which was not certified by peer review) is the author/funder, who has granted bioRxiv a license to display the preprint in perpetuity. It is made available under aCC-BY 4.0 International license.

Table S5. Full GAMM results of model comparisons from caudate.

Caudate											
	**Parametric coefficients**						**Parametric coefficients**				
		Estimate	S.E.	*t*	*p*			Estimate	S.E.	*t*	*p*
	Intercept	0.87	0.01	94.99	< 0.001***		Intercept	0.87	0.01	93.65	< 0.001***
	Sex	0.002	0.01	0.18	0.86		Sex	0.001	0.01	0.10	0.9
	Visit	0.03	0.005	5.61	< 0.001***		Visit	0.03	0.005	5.53	< 0.001***
	**Approx. significance of smooth terms**						**Approx. significance of smooth terms**				
**Model 1**		edf	Ref. edf	*F*	*p*	**Model 2**		edf	Ref. edf	*F*	*p*
	Age	1.92	1.96	5.96	0.003**		Age	1.92	1.96	5.70	0.004**
	FSS	-	-	-	-		FSS	1.31	1.45	0.25	0.59
	MB	-	-	-	-		MB	-	-	-	-
	MF	-	-	-	-		MF	-	-	-	-
	**Random effects**						**Random effects**				
		sd		95% CI				*sd*		95% CI	
	Intercept	0.054		[0.045, 0.064]			Intercept	0.054		[0.048, 0.064]	
	Residuals	0.048		[0.042, 0.055]			Residuals	0.048		[0.042, 0.055]	
	**Parametric coefficients**						**Parametric coefficients**				
		Estimate	S.E.	*t*	*p*			Estimate	S.E.	*t*	*p*
	Intercept	0.87	0.01	93.08	< 0.001***		Intercept	0.87	0.01	92.92	< 0.001***
	Sex	0.001	0.01	0.08	0.93		Sex	0.0004	0.01	0.05	0.96
	Visit	0.03	0.005	5.43	< 0.001***		Visit	0.03	0.005	5.42	< 0.001***
	**Approx. significance of smooth terms**						**Approx. significance of smooth terms**				
**Model 3**		*edf*	Ref. edf	*F*	*P*	**Model 4**		*edf*	Ref. edf	*F*	*p*
	Age	1.92	1.96	5.66	< 0.001***		Age	1.92	1.96	5.66	0.004**
	FSS	1.31	1.45	0.10	0.81		FSS	1.27	1.39	0.09	0.79
	MB	1	1	0.27	0.60		MB	1	1	0.33	0.57
	MF	-	-	-	-		MF	1	1	0.12	0.73
	**Random effects**						**Random effects**				
		sd		95% CI				*sd*		95% CI	
	Intercept	0.053		[0.045, 0.064]			Intercept	0.054		[0.046, 0.064]	
	Residuals	0.048		[0.042, 0.055]			Residuals	0.048		[0.042, 0.055]	

Note. Edf = effective degrees of freedom. FSS = first-stage stay. MB = model-based. MF = model-free. * *p* is significant at Bonferroni-adjusted α of 0.0125.

bioRxiv preprint doi: https://doi.org/10.1101/2025.01.06.631527; this version posted January 6, 2025. The copyright holder for this preprint (which was not certified by peer review) is the author/funder, who has granted bioRxiv a license to display the preprint in perpetuity. It is made available under aCC-BY 4.0 International license.

Table S6. Full GAMM results of model comparisons from NAcc.

NAcc

Model 1

**Parametric coefficients**	Estimate	S.E.	*t*	*p*
Intercept	0.88	0.02	48.18	< 0.001***
Sex	-0.04	0.02	-2.16	0.03*
Visit	0.03	0.01	2.69	0.008**
**Approx. significance of smooth terms**	edf	Ref. edf	*F*	*p*
Age	1	1	18.80	< 0.001***
FSS	-	-	-	-
MB	-	-	-	-
MF	-	-	-	-
**Random effects**	*sd*		95% CI	
Intercept	0.114		[0.097, 0.135]	
Residuals	0.091		[0.080, 0.106]	

Model 2

**Parametric coefficients**	Estimate	S.E.	*t*	*p*
Intercept	0.88	0.02	47.31	< 0.001***
Sex	-0.04	0.02	-1.99	0.048*
Visit	0.03	0.01	2.76	0.007**
**Approx. significance of smooth terms**	edf	Ref. edf	*F*	*p*
Age	1	1	18.84	< 0.001***
FSS	1.42	1.58	0.60	0.64
MB	-	-	-	-
MF	-	-	-	-
**Random effects**	*sd*		95% CI	
Intercept	0.114		[0.097, 0.135]	
Residuals	0.092		[0.080, 0.106]	

Model 3

**Parametric coefficients**	Estimate	S.E.	*t*	*p*
Intercept	0.88	0.02	46.97	< 0.001***
Sex	-0.04	0.02	-1.98	0.049*
Visit	0.03	0.01	2.74	0.007**
**Approx. significance of smooth terms**	*edf*	Ref. edf	*F*	*P*
Age	1	1	18.79	< 0.001***
FSS	1.43	1.58	0.46	0.69
MB	1	1	0.01	0.93
MF	-	-	-	-
**Random effects**	*sd*		95% CI	
Intercept	0.114		[0.096, 0.135]	
Residuals	0.092		[0.080, 0.106]	

Model 4

**Parametric coefficients**	Estimate	S.E.	*t*	*p*
Intercept	0.88	0.02	46.84	< 0.001***
Sex	-0.04	0.02	-1.97	0.051
Visit	0.03	0.01	2.74	0.007**
**Approx. significance of smooth terms**	*edf*	Ref. edf	*F*	*p*
Age	1	1	18.74	< 0.001***
FSS	1.43	1.59	0.47	0.69
MB	1	1	0.01	0.91
MF	1	1	0.01	0.93
**Random effects**	*sd*		95% CI	
Intercept	0.114		[0.096, 0.135]	
Residuals	0.092		[0.080, 0.106]	

Note. Edf = effective degrees of freedom. FSS = first-stage stay. MB = model-based. MF = model-free. * p is significant at Bonferroni-adjusted α of 0.0125.

bioRxiv preprint doi: https://doi.org/10.1101/2025.01.06.631527; this version posted January 6, 2025. The copyright holder for this preprint (which was not certified by peer review) is the author/funder, who has granted bioRxiv a license to display the preprint in perpetuity. It is made available under aCC-BY 4.0 International license.

Table S7. Full GAMM results of model comparisons from globus pallidus.

	**Globus Pallidus**										
	**Parametric coefficients**						**Parametric coefficients**				
		Estimate	S.E.	*t*	*p*			Estimate	S.E.	*t*	*p*
	Intercept	0.58	0.01	89.56	< 0.001***		Intercept	0.58	0.01	88.16	< 0.001***
	Sex	-0.004	0.01	-0.64	0.52		Sex	-0.003	0.01	-0.48	0.64
	Visit	-0.004	0.003	-1.10	0.27		Visit	-0.003	0.003	-1.03	0.031
	**Approx. significance of smooth terms**						**Approx. significance of smooth terms**				
**Model 1**		edf	Ref. edf	*F*	*p*	**Model 2**		edf	Ref. edf	*F*	*p*
	Age	1.19	1.26	44.67	< 0.001***		Age	1.05	1.08	50.62	< 0.001***
	FSS	-	-	-	-		FSS	1	1	0.73	0.40
	MB	-	-	-	-		MB	-	-	-	-
	MF	-	-	-	-		MF	-	-	-	-
	**Random effects**						**Random effects**				
		sd		95% CI				*sd*		95% CI	
	Intercept	0.040		[0.340, 0.047]			Intercept	0.040		[0.034, 0.048]	
	Residuals	0.033		[0.029, 0.038]			Residuals	0.033		[0.028, 0.038]	
	**Parametric coefficients**						**Parametric coefficients**				
		Estimate	S.E.	*t*	*p*			Estimate	S.E.	*t*	*p*
	Intercept	0.58	0.01	87.63	< 0.001***		Intercept	0.58	0.01	87.46	< 0.001***
	Sex	-0.003	0.01	-0.49	0.63		Sex	-0.004	0.01	-0.51	0.61
	Visit	-0.004	0.003	-1.06	0.29		Visit	-0.004	0.003	-1.05	0.30
	**Approx. significance of smooth terms**						**Approx. significance of smooth terms**				
**Model 3**		*edf*	Ref. edf	*F*	*P*	**Model 4**		*edf*	Ref. edf	*F*	*P*
	Age	1.06	1.09	49.89	< 0.001***		Age	1.09	1.12	48.17	< 0.001***
	FSS	1	1	0.82	0.37		FSS	1	1	0.65	0.42
	MB	1	1	0.15	0.70		MB	1	1	0.18	0.68
	MF	-	-	-	-		MF	1.21	1.39	0.65	0.42
	**Random effects**						**Random effects**				
		sd		95% CI				*sd*		95% CI	
	Intercept	0.040		[0.034, 0.048]			Intercept	0.040		[0.034, 0.048]	
	Residuals	0.033		[0.028, 0.038]			Residuals	0.033		[0.028, 0.038]	

Note. Edf = effective degrees of freedom. FSS = first-stage stay. MB = model-based. MF = model-free. * *p* is significant at Bonferroni-adjusted α of 0.0125.

bioRxiv preprint doi: https://doi.org/10.1101/2025.01.06.631527; this version posted January 6, 2025. The copyright holder for this preprint (which was not certified by peer review) is the author/funder, who has granted bioRxiv a license to display the preprint in perpetuity. It is made available under aCC-BY 4.0 International license.

S.4 Supplementary results from section 3.5

Detailed summary tables from GAMMs assessing whether developmental trajectories of tissue iron in the putamen varied with first-stage stay and model-based behavior (Table S8). Both time-varying parameters were significant, indicating that developmental trajectories of putamen tissue iron differed at varying levels of behavioral performance.

Table S8. Time-varying parameter modeling results.

**Putamen**				
**Parametric coefficients**				
	**Estimate**	**S.E.**	***t***	***p***
Intercept	0.87	0.01	117.63	< 0.001***
Sex	-0.02	0.01	-3.10	0.002**
Visit	0.02	0.004	4.23	< 0.001***
**Approx. significance of smooth terms**				
	**edf**	**Ref. df**	***F***	***p***
Age	1	1	91.02	< 0.001***
Age:First stage stay	2	2	4.34	0.014*
Age:Model based	2	2	4.36	0.014**
**Random effects**				
	sd		**95% CI**	
Intercept	0.045		[0.04, 0.05]	
Residuals	0.037		[0.03, 0.04]	

Note. Edf = effective degrees of freedom. * = $p < 0.05$, ** = $p < 0.01$, *** = $p < 0.001$.

bioRxiv preprint doi: https://doi.org/10.1101/2025.01.06.631527; this version posted January 6, 2025. The copyright holder for this preprint (which was not certified by peer review) is the author/funder, who has granted bioRxiv a license to display the preprint in perpetuity. It is made available under aCC-BY 4.0 International license.

S.5 Supplementary results from section 3.6

Detailed summary tables from GAMMs fit for the specificity analyses are presented in Supplementary Table S9.

Table S9. GAMM results characterizing developmental trajectories of putamen nT2*w according to CIC atlas.

ROI	Parametric coefficients				
**Ant. dorsal putamen**		Estimate	S.E.	*t*	*p*
	Intercept	0.87	0.01	104.19	< 0.001*
	Sex	-0.02	0.01	-2.74	0.006*
	Visit	0.03	0.005	5.67	< 0.001*
	**Approx. significance of smooth terms**				
		edf	Ref. edf	*F*	*p*
	Age	1	1	69.71	< 0.001*
	Age:FSS	2	2	2.27	0.11
	Age:MB	2	2	2.27	0.11
	**Random effects**				
		sd		95% CI	
	Intercept	0.044		[0.035, 0.055]	
	Residuals	0.045		[0.039, 0.053]	
**Post. dorsal putamen**	**Parametric coefficients**				
		Estimate	S.E.	*t*	*p*
	Intercept	0.89	0.01	104.69	< 0.001*
	Sex	-0.02	0.01	-1.84	0.07
	Visit	0.01	0.005	2.31	0.02
	**Approx. significance of smooth terms**				
		edf	Ref. edf	*F*	*P*
	Age	1	1	70.79	< 0.001*
	Age:FSS	2.63	2.77	3.37	0.02
	Age:MB	2	2	6.39	0.002*
	**Random effects**				
		sd		95% CI	
	Intercept	0.046		[0.038, 0.057]	
	Residuals	0.046		[0.040, 0.053]	

ROI	Parametric coefficients				
**Ant. ventral putamen**		Estimate	S.E.	*t*	*p*
	Intercept	0.88	0.01	98.79	< 0.001*
	Sex	-0.04	0.01	-4.03	< 0.001*
	Visit	0.01	0.004	2.59	0.01*
	**Approx. significance of smooth terms**				
		edf	Ref. edf	*F*	*p*
	Age	1	1	101.55	< 0.001*
	Age:FSS	2	2	2.71	0.07
	Age:MB	2	2	2.12	0.12
	**Random effects**				
		sd		95% CI	
	Intercept	0.043		[0.049, 0.053]	
	Residuals	0.045		[0.039, 0.053]	
**Post. ventral putamen**	**Parametric coefficients**				
		Estimate	S.E.	*t*	*p*
	Intercept	0.88	0.01	79.07	< 0.001*
	Sex	-0.02	0.01	-1.99	0.048
	Visit	0.003	0.01	0.47	0.64
	**Approx. significance of smooth terms**				
		edf	Ref. edf	*F*	*p*
	Age	1.66	1.77	49.22	< 0.001*
	Age:FSS	2.79	2.89	3.84	0.0124*
	Age:MB	2	2	4.55	0.0118*
	**Random effects**				
		sd		95% CI	
	Intercept	0.06		[0.05, 0.07]	
	Residuals	0.06		[0.05, 0.07]	

Note. Edf = effective degrees of freedom. Ant. = anterior. Post. = posterior. FSS = first-stage stay. MB = model-based. * *p* is significant at Bonferroni-adjusted α of 0.0125.

bioRxiv preprint doi: https://doi.org/10.1101/2025.01.06.631527; this version posted January 6, 2025. The copyright holder for this preprint (which was not certified by peer review) is the author/funder, who has granted bioRxiv a license to display the preprint in perpetuity. It is made available under aCC-BY 4.0 International license.

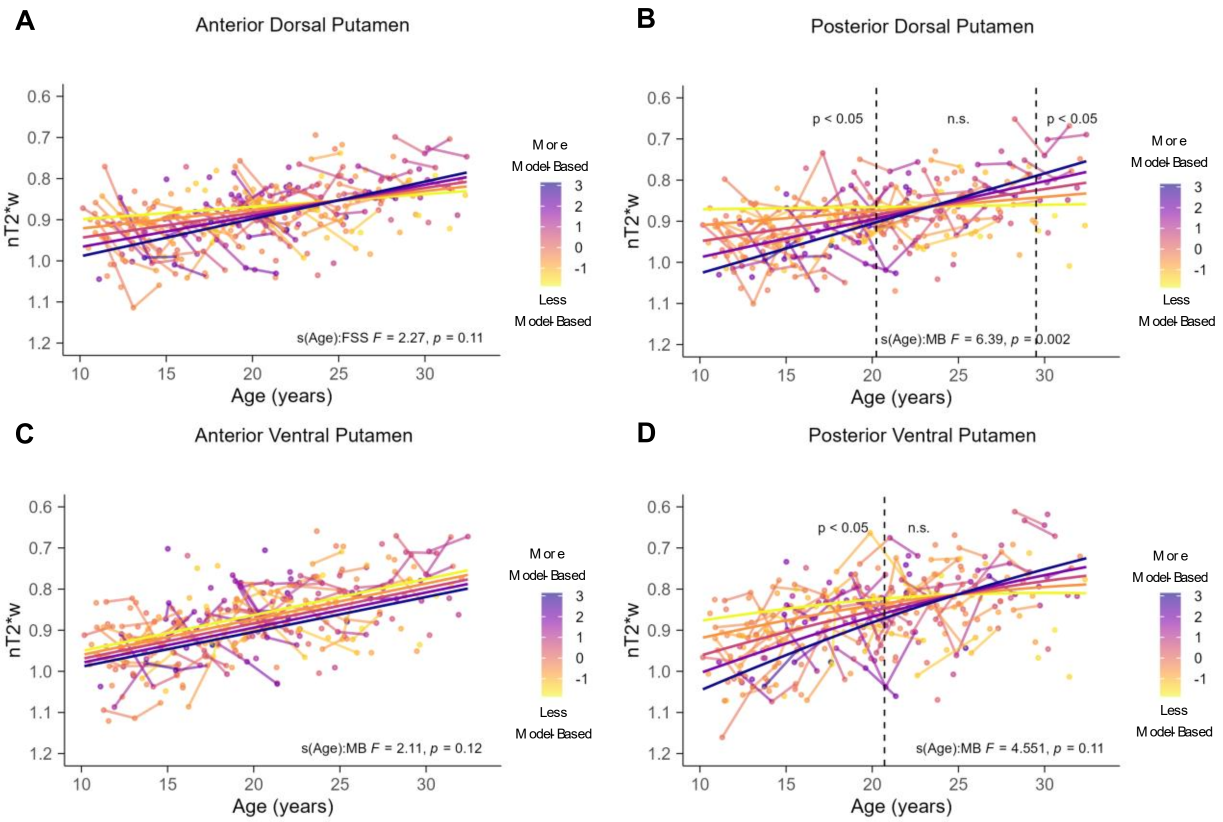

Figure S1. Time-varying parameter model results focusing on model-based behavior. In all panels, the y-axis is reversed such that lower values reflect more tissue iron. The color of the points represents model-based z-scores with darker colors indicating more model-based behavior. There were no significant associations between age and model-based behavior on anterior dorsal putamen tissue iron (A) or anterior ventral putamen tissue iron (C). (B) Model-based behavior moderated the association between age and posterior dorsal putamen tissue iron. The dashed vertical lines at 20.25 and 29.63 years old reflects the age boundaries where the 95% CI of the derivative does not include 0, indicating significance. (D) Model-based behavior moderated the association between age and posterior ventral putamen tissue iron. The dashed line at 20.71 years old reflects the age boundary where the 95% CI of the derivate does not include 0, indicating significance.

bioRxiv preprint doi: https://doi.org/10.1101/2025.01.06.631527; this version posted January 6, 2025. The copyright holder for this preprint (which was not certified by peer review) is the author/funder, who has granted bioRxiv a license to display the preprint in perpetuity. It is made available under aCC-BY 4.0 International license.

S.6 Supplementary Behavioral Results

S.6.1 Multilevel logistic regression with continuous age

We tested for age related differences in the recruitment of each strategy using a continuous age term and its interaction terms, similar to the analyses conducted by Decker and colleagues (2016). The multilevel logistic regression model was specified as follows (using R syntax):

glmer(firststagestay ~ 1 + age_z*transition*reward + (transitions*reward|id:visit)) (S1)

where the model was fit using the optimizer Bound Optimization by Quadratic Approximation (BOBYQA; Powell, 2009). Results from the multilevel logistic regression model are presented in Table S10. The effects of transition type and reward on the probability of a first-stage stay (FSS) faceted by age groups are presented in Supplementary Figure S2.

Table S10. Multilevel logistic regression results

**Fixed effects**	**Estimate**	**S.E.**
Intercept	1.51***	0.06
Age	0.53***	0.06
Transition type	0.05*	0.02
Reward	0.38***	0.02
**Interactions**		
Age x Transition type	0.08***	0.02
Age x Reward	0.16***	0.02
Transition x Reward	0.42***	0.03
Age x Transition Type x Reward	0.12***	0.03

Note. 82,024 observations nested in 320 visits nested in 217 participants. * = $p < 0.05$. *** = $p < 0.001$

bioRxiv preprint doi: https://doi.org/10.1101/2025.01.06.631527; this version posted January 6, 2025. The copyright holder for this preprint (which was not certified by peer review) is the author/funder, who has granted bioRxiv a license to display the preprint in perpetuity. It is made available under aCC-BY 4.0 International license.

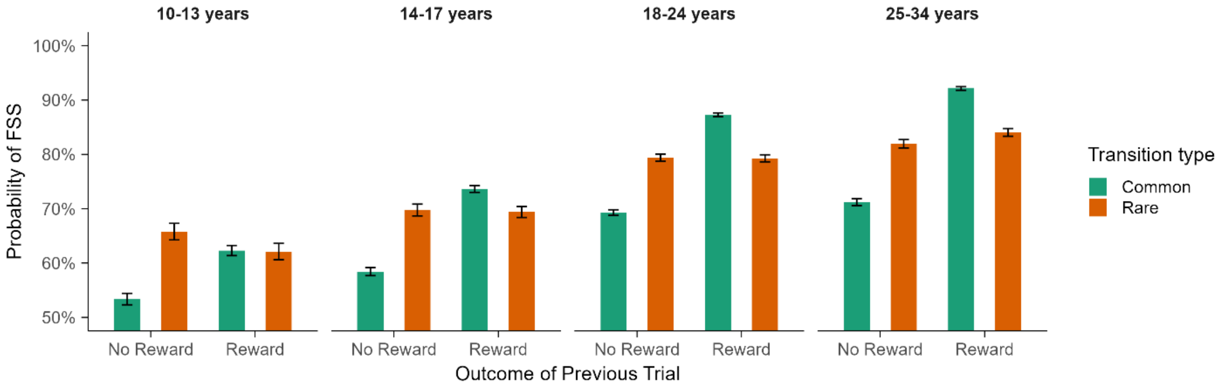

Figure S2. Probability of first-stage stays, averaged across participants and binned by representative age groups. Error bars represent +/- 1 SEM. Across all age groups, there is evidence of participants using both model-based and model-free strategies.

The main effects of age, transition type, and reward were significant, indicating that as age increased, the probability of a FSS increased (Figure S3 Panel A.), the probability of a FSS was greater after common transitions (Figure S3 Panel B.), and the probability of a FSS was greater after earning a reward (Figure S3 Panel C.). All two-way interactions were significant. We found that transition type moderated the association between age and FSS (Figure S3 Panel D), such that as age increased common transitions were associated with more FSS. We found that reward moderated the association between age and FSS (Figure S3 Panel E), such that in young participants the difference between receiving a reward and not were less associated with FSS, in older participants that difference was more pronounced, in that older adults had more FSS after receiving a reward. We also found that reward moderated the association between transition type and FSS (Figure S3, Panel F), such that the highest probability of a FSS occurred after being rewarded after a common transition. We also observed a significant three-way interaction (Figure S4). After rare transitions, there were no differences between earning and not earning a reward across the entire range of age, whereas after common transitions, the difference between receiving a reward and not were less associated with FSS, compared to older participants where receiving a reward was associated with more FSS. Taken together, these findings suggest that model free (main effect of reward) and model based (transition type x reward interaction) behaviors are evident in the full sample, albeit to differing degrees. Adults appear to be using all strategies at a higher level than adolescents.

bioRxiv preprint doi: https://doi.org/10.1101/2025.01.06.631527; this version posted January 6, 2025. The copyright holder for this preprint (which was not certified by peer review) is the author/funder, who has granted bioRxiv a license to display the preprint in perpetuity. It is made available under aCC-BY 4.0 International license.

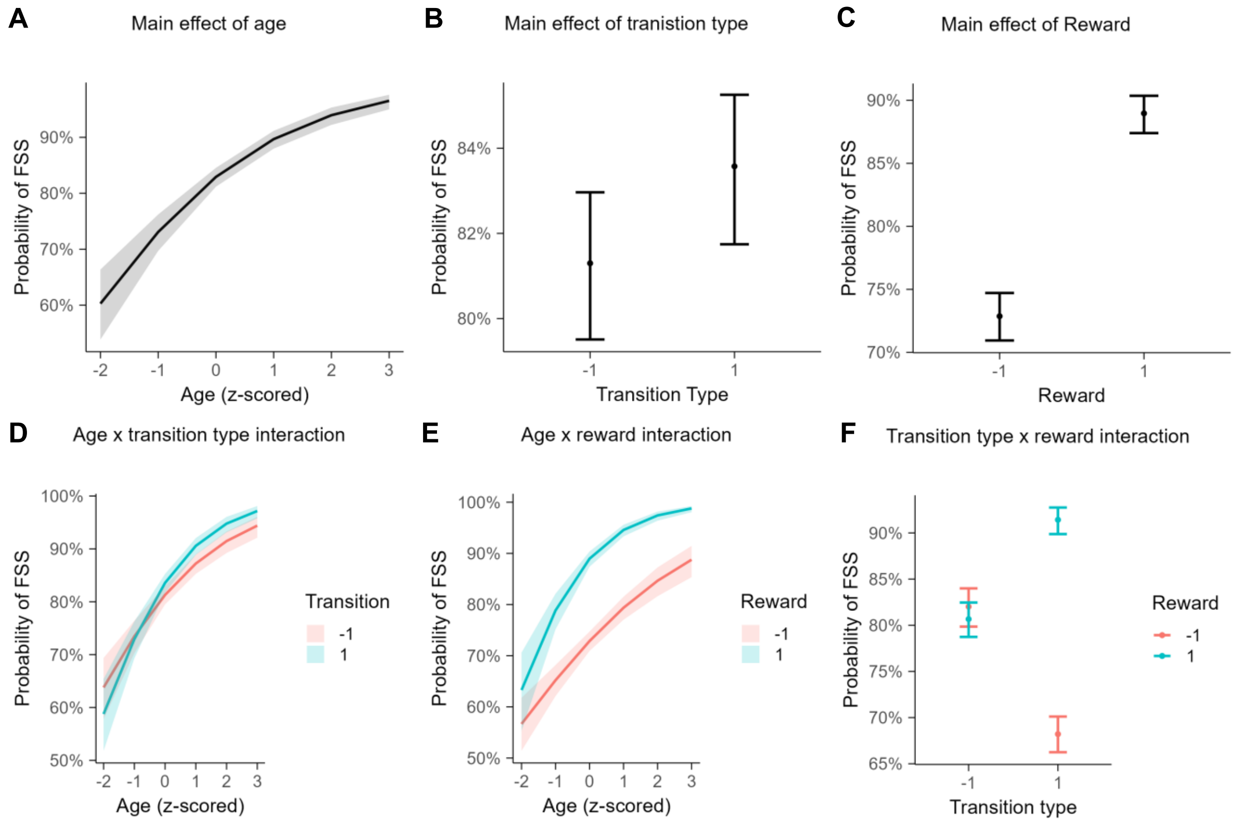

Figure S3. Marginal effects plots from the multilevel logistic regression model with continuous age. (A.) We observed a main effect of age, such that older participants had a higher probability of a FSS compared to younger participants. (B.) We observed a main effect of transition type, such that common transitions were associated with a higher probability of a FSS compared to rare transitions. (C.) We observed a main effect of reward (i.e., model-free behavior), such that receiving a reward was associated with a higher probability of a FSS compared to not being rewarded. (D.) We observed an age by transition type interaction, such that the probability of a FSS was greater after a common transition compared to a rare transition for adults but not for adolescents. (E.) We observed an age by reward interaction, such that the difference in the probability of a FSS after receiving versus not receiving a reward was the most pronounced for older participants compared to younger participants. (F.) We observed a transition type by reward interaction (i.e., model-based behavior), such that after rare transitions, there was minimal differences in the probability of a FSS after receiving verses not receiving a reward. For common transitions, the probability of a FSS was the highest after receiving a reward.

bioRxiv preprint doi: https://doi.org/10.1101/2025.01.06.631527; this version posted January 6, 2025. The copyright holder for this preprint (which was not certified by peer review) is the author/funder, who has granted bioRxiv a license to display the preprint in perpetuity. It is made available under aCC-BY 4.0 International license.

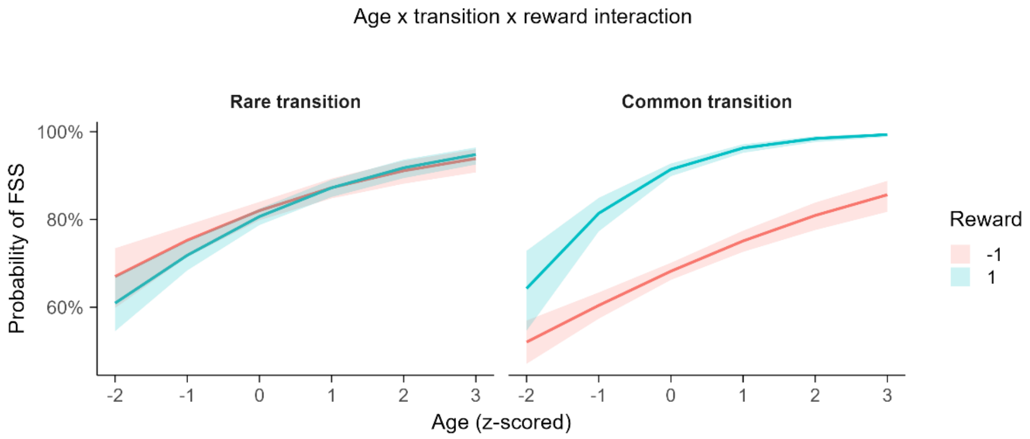

Figure S4. Marginal effects plot of the three-way interaction from the multilevel logistic regression model with continuous age. The probability of a FSS after a rare transition increased with age, but was not different depending on whether participants earned a reward or not. The probability of a FSS after a common transition also increased with age. However, the age increase in FSS was pronounced after being rewarded compared to not being rewarded.

S.6.2 Relation between knowledge of task structure and learning

Similar to other developmental studies using the sequential decision-making task (Decker et al., 2016; Nussenbaum et al., 2020; Potter et al., 2017), we assessed whether participant responses reflected knowledge of task structure, and whether knowledge of task structure was related to model-based control.

To assess participants' knowledge of task structure, we examined whether participants' response times when making second-stage choices were affected by the type of transition they experienced. If participants were unaware of the task structure, we would expect no difference in RTs following common versus rare transitions. Conversely, if they understood the transition structure, we would anticipate slower responses after rare transitions. A linear mixed-effect model was used to examine these associations (see Table S11 and Figure S5A). Results suggest that participants were slower after rare transitions compared to common ones (β = -41.27, SE = 1.47, $p < 0.001$), that older participants made faster choices (β = -31.38, SE = 8.49, $p < 0.001$) and that the effect of transition type on reaction times increased with increasing age (β = -20.98, SE = 1.47, $p < 0.001$).

To assess whether knowledge of task structure was related to model-based control, we fit an additional linear multilevel model where we computed a reaction time difference score for the second stage choice by subtracting each participant's mean reaction time after common transitions from each participant's mean reaction time after rare transitions. Then, we regressed the model-based coefficient on age, the mean difference in reaction times during the second stage choice, and their interaction. We found the reaction time difference scores were associated with model-based control (β = 0.15, SE = 0.02, $p < 0.001$), indicating that slower reaction times after rare transitions were associated with more model-based control. We did not observe a significant reaction time difference by age interaction when we treated age as a continuous variable. Full results are presented in Table S12 and Figure S5B. Taken together, these results suggest that

bioRxiv preprint doi: https://doi.org/10.1101/2025.01.06.631527; this version posted January 6, 2025. The copyright holder for this preprint (which was not certified by peer review) is the author/funder, who has granted bioRxiv a license to display the preprint in perpetuity. It is made available under aCC-BY 4.0 International license.

participants did understand the transition structure of the task. However, early adolescents do not appear to be integrating this information to the extent that mid/late adolescents and adults do. Our behavioral results are generally consistent with other research groups who have administered this task to developmental samples.

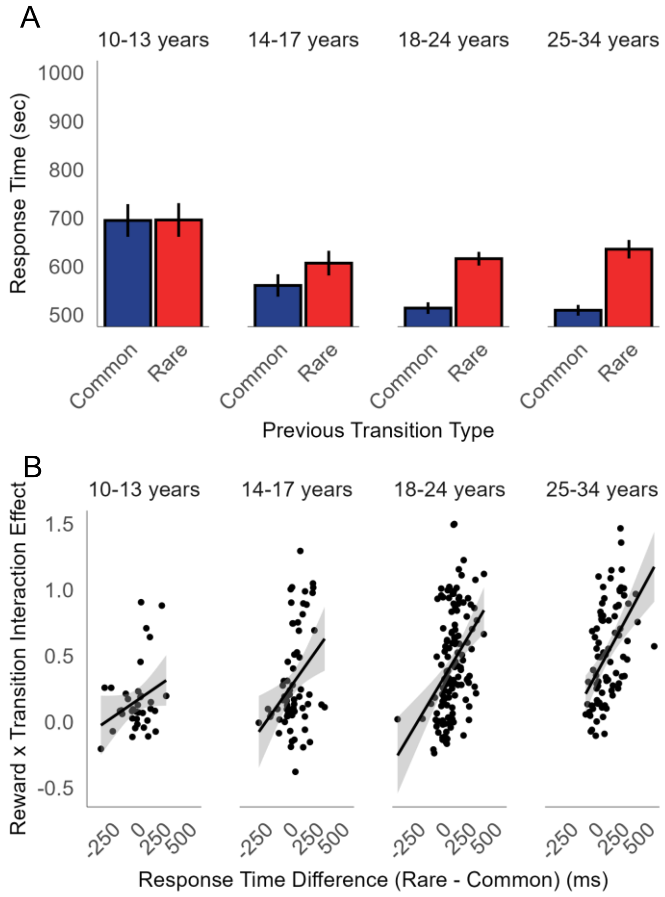

Figure S5. Second-stage response time results. (A) Bar graphs depicting response times for choices during the second-stage as a function of the previous transition type for each age group. Error bars reflect ± 1 *SEM*. On average, participants were slower during their second-stage choices following rare transitions compared to common transitions. This difference in response times between transition types increased across age. (B) Scatter plots depicting the relationship between the difference in response times between rare and common transitions and the reward x transition interaction effect (i.e., model-based term) for each age group. On average, participants with larger response times after rare transitions compared to common transitions had a larger reward x transition interaction effect. This effect increased across age. These results suggest that knowledge of task structure was related to participants' use of model-based control.

bioRxiv preprint doi: https://doi.org/10.1101/2025.01.06.631527; this version posted January 6, 2025. The copyright holder for this preprint (which was not certified by peer review) is the author/funder, who has granted bioRxiv a license to display the preprint in perpetuity. It is made available under aCC-BY 4.0 International license.

Table S11. Linear multilevel modeling coefficients indicating the effect of age and transition type on second-stage choice reaction times

**Fixed Effects**	**Estimate**	**SE**	***p***
Intercept	583.55	8.50	< 0.001
Age	-31.38	8.49	< 0.001
Transition type	-41.27	1.47	< 0.001
Age x Transition type	-20.98	1.47	< 0.001
**Random Effects**	***sd***	**95% CI**	
Intercept	149.70	[138.08, 161.89]	
Residuals	342.4	[340.48, 344.23]	

Table S12. Linear multilevel modeling coefficients indicating the effect of age and RT differences on model-based control

**Fixed Effects**	**Estimate**	**SE**	***p***
Intercept	0.38	0.02	< 0.001
Age	0.07	0.02	0.0014
RT difference	0.15	0.02	< 0.001
Age x RT difference	0.01	0.02	0.48
**Random Effects**	***sd***	**95% CI**	
Intercept	0.21	[0.16, 0.26]	
Residuals	0.26	[0.23, 0.30]	

Note. RT = Reaction Time

S.7 Reinforcement learning model details

The primary purpose of fitting RL models to the behavioral data was to compare the first-stage stay measure used in the current study to a commonly estimated perseverance parameter that is estimated in some RL models. Briefly, the perseverance parameter tracks the tendency to repeat a first-stage choice regardless of outcome (i.e., outcome insensitive, which is indicative of habits). Thus, if the first-stage stay measure is tracking habitual behavior, then it should correlate with the perseverance parameter. The following section describes the set of RL models used for this comparison in more detail.

S.7.1 hBayesDM

Hierarchical Bayesian analyses (HBA) were conducted using the hierarchical Bayesian Decision-Making (hBayesDM) package for R (Ahn et al., 2017) that uses rstan (version 2.32.6) for implementation. In Bayesian statistics, we start with prior beliefs (known as prior distributions) about the model parameters. These priors are updates into posterior distributions based on the data (i.e., trial-by-trial choice and outcomes during the two-stage task) by applying Bayes rule (Kruschke, 2014). In a HBA, group or hyperparameters are introduced in addition to

bioRxiv preprint doi: https://doi.org/10.1101/2025.01.06.631527; this version posted January 6, 2025. The copyright holder for this preprint (which was not certified by peer review) is the author/funder, who has granted bioRxiv a license to display the preprint in perpetuity. It is made available under aCC-BY 4.0 International license.

the individual-level parameters which leads to shrinkage effects (Gelman et al., 1995). Shrinkage effects in a HBA occur when each individual's estimates influence the group's overall estimates, which then feedback to refine the estimates for each individual. A result of shrinkage effects is that individual estimates are often more stable and reliable with this approach, because similarities among participants are informed by the group parameters. Posterior inference for hBayesDM models use a Markov chain Monte Carlo (MCMC) sampling scheme called Hamiltonian Monte Carlo (HMC; (Carpenter et al., 2017). A full description of hBayesDM can be found in Ahn et al. (2017).

S.7.2 Two-stage sequential decision-making parameter descriptions

The hBayesDM packages come with three pre-programmed RL models based on RL models described in Daw et al. (2011) and Wunderlich et al., (2012). Each model differs in the number of free parameters that are estimated (either 4 parameters, 6 parameters, or 7 parameters). All RL models contain the following free parameters (α, β, π, w):

The alpha (α) parameter (learning rate) determines how quickly an agent updates their expectation based on the prediction error (e.g., the difference between expected and actual outcomes) at both the first and second stages. A higher learning rate means the agent rapidly adapts to new information, while a lower learning rate indicates a more conservative updating of beliefs. The beta (β) parameter (inverse temperature) controls the balance between exploration and exploitation in decision-making. A higher beta value means the agent is more deterministic in their choices, favoring the option with the highest expected value, implying more exploitation. A lower beta suggests greater randomness in choice, implying more exploration. The pi (π) parameter (perseverance) reflects the tendency of the agent to repeat a previous choice regardless outcome. It captures how "sticky" the agent's bias is towards a previously chosen action. Higher values indicate a stronger tendency to persevere with the same choice. The W (w) parameter (model-based weight) represents the weight given to model-based versus model-free behavior. Model-based behavior involves planning and using a cognitive map of the task structure to make decisions, while model-free behavior relies on cached values from past experiences. A higher w indicates greater reliance on model-based strategies, whereas a lower w suggests more dependence on model-free learning.

The only difference between the 4-parameter model and the 6-parameter model is that α and β are estimated for each stage of the task (e.g., α stage 1/ α stage 2, β stage 1/ β stage 2). For the 7-parameter model, a lambda (λ) free parameter is estimated. Lambda is used in temporal difference learning to determine how much of the prediction error is assigned to past states and actions. It influences the extent to which past experiences (not just the most recent one) are updated based on new information. A higher lambda means that more distant past experiences are considered when updating value.

S.7.3 Computational model description

For the full description of the computational model, see Daw et al., (2011). hBayesDM code for the two-stage sequential decision-making task is from the publicly available GitHub repository (https://github.com/ccs-lab/hBayesDM). Briefly, the task has three states (first stage: s_A; second stage: s_B and s_C) and two actions (a_A and a_B). The temporal difference learning algorithm learns a state-action value function $Q_{s,a}$ by mapping each state and action to its

bioRxiv preprint doi: https://doi.org/10.1101/2025.01.06.631527; this version posted January 6, 2025. The copyright holder for this preprint (which was not certified by peer review) is the author/funder, who has granted bioRxiv a license to display the preprint in perpetuity. It is made available under aCC-BY 4.0 International license.

expected future value by an update rule for stage *i* for each trial *t* according to (notation is from Daw et al. (2011):

$$Q_{TD}(s_{i,t}, a_{i,t}) = Q_{TD}(s_{i,t}, a_{i,t}) + \alpha_i \delta_{i,t}$$

where

$$\delta_{i,t} = r_{i,t} + Q_{TD}(s_{i+1}, a_{i+1}) - Q_{TD}(s_{i,t}, a_{i,t})$$

and α_i is the learning rate parameter. $\delta_{i,t}$ reflects the reward prediction error which is driven by the second-stage value only (because $r_{i,t} = 0$). The λ parameter effects first stage action by the second stage reward prediction error according to:

$$Q_{TD}(s_{1,t}, a_{1,t}) = Q_{TD}(s_{1,t}, a_{1,t}) + \alpha_1 \lambda \delta_{2,t}$$

The w parameter is introduced to connect values to choices by a weighted sum of model-based and model-free values according to:

$$Q_{net}(s_A, a_j) = wQ_{MB}(s_A, a_j) + (1 - w)Q_{TD}(S_A, a_j)$$

Finally, the probability of a choice is calculated via a softmax function as:

$$P(a_{i,t} = a | s_{i,t}) = \frac{\exp(\beta_i[Q_{net}(s_{i,t}, a) + \pi * rep(a)])}{\Sigma_{a'} \exp(\beta_i[Q_{net}(s_{i,t}, a') + \pi * rep(a')])}$$

where β_i is the inverse temperature parameter that control how deterministic or stochastic the choices are and π is the perseverance parameter that controls for how sticky first stage choices are.

S.7.4 Model selection

We fit the three described models on the behavioral data, each with 4 chains, 1000 burn-in samples, and 3000 samples. Model selection was conducted using Leave-One-Out Information Criterion (LOOIC) and Widely Applicable Information Criterion (WAIC; see Vehtari et al., 2017 for details). Lower LOOIC and WAIC indicate better model fit. The 7-paramter model was found to be the winning model and was used for further analyses.

Table S11. Global fit statistics used for model selection

Model	LOOIC	WAIC
4-parameter model	165142.20	164181.40
6-parameter model	163580.30	162760.00
7-parameter model	**163545.50**	**162754.00**

Note. Lowest LOOIC/WAIC are made bold.

S.7.5 Assessing model convergence

We examined $\hat{R}$ and visually diagnosed MCMC performance to assess model convergence. $\hat{R}$ values for the hyper parameters were all close to 1, indicating convergence ($\hat{R}$ 1 – 1.01). For individual parameter estimates (including the posterior prediction estimates), only five individual parameters had an $\hat{R}$ value that exceeded 1.04. For the visual diagnosis, we checked whether MCMC samples were well mixed and converged to stationary distributions.

bioRxiv preprint doi: https://doi.org/10.1101/2025.01.06.631527; this version posted January 6, 2025. The copyright holder for this preprint (which was not certified by peer review) is the author/funder, who has granted bioRxiv a license to display the preprint in perpetuity. It is made available under aCC-BY 4.0 International license.

S.7.6 Bivariate association between first-stage stay and perseverance parameters

We assessed the bivariate association between first-stage stay behavior derived from the multilevel logistic regression and perseverance parameter from the best fitting RL model. If first-stage stay behavior is indicative of habitual behavior, then it should be correlate with the perseverance parameter. Additionally, the perseverance parameter should have a similar developmental trajectory compared to the first-stage stay measure. Results from the supplemental analysis found that the correlation between first-stage stay and perseverance was high (Figure S6 A; $r = 0.91$). Both measures also had similar developmental trajectory patterns, with increases across the age range (Figure S6 B). Thus, it is reasonable to suggest that first-stage stay is tracking habitual responding during the two-stage sequential decision-making task.

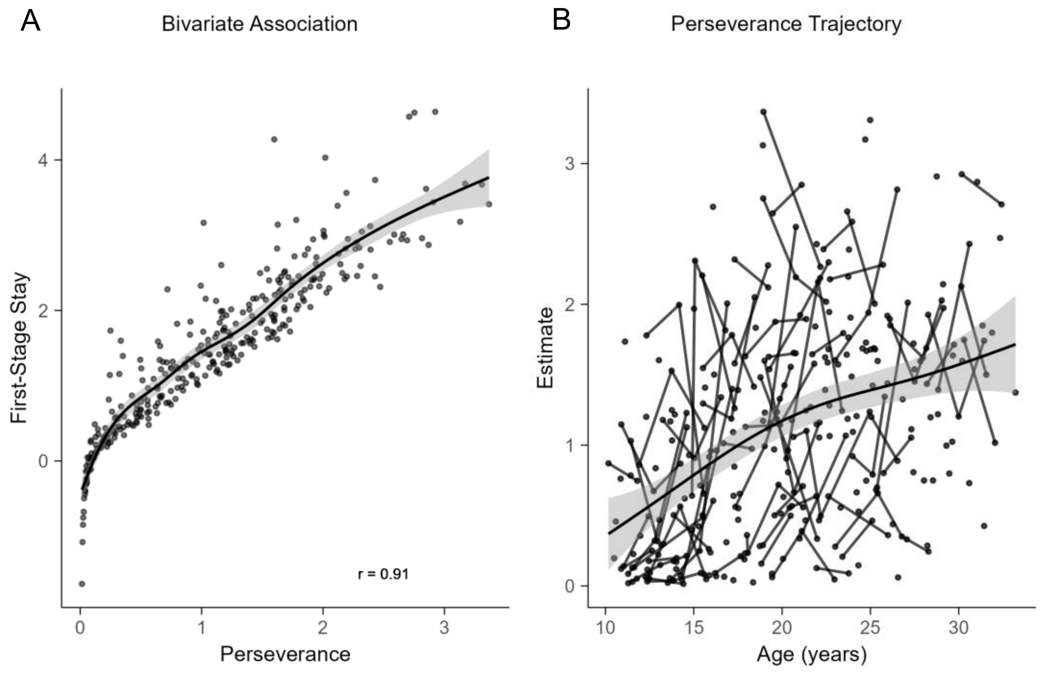

Figure S6. Plots comparing the first-stage stay measure to the perseverance parameter. (A) The bivariate correlation between perseverance and first-stage stay is large ($r = 0.91$) indicating that they measure a similar latent construct (i.e., habitual responding). (B & C) We observed similar age trajectories for the first-stage stay measure and the perseverance parameter.
